# Reactive oxygen species, oxidative signaling and the regulation of photosynthesis

**DOI:** 10.1016/j.envexpbot.2018.05.003

**Published:** 2018-10

**Authors:** Christine H. Foyer

**Affiliations:** Centre for Plant Sciences, School of Biology, Faculty of Biological Sciences, University of Leeds, LS2 9JT, United Kingdom

**Keywords:** Ascorbate, Antioxidants, Acclimation, Chloroplast-to-nucleus retrograde signaling, Epigenetics, Glutathione, High Light, Hydrogen peroxide, Mehler reaction, MSH1 pathway, NPQ, Peroxiredoxins, Photodamage, Photoinhibition, Photosystem II, Photosynthesis, Post translational modifications, Reactive oxygen species, Redox signaling, Singlet Oxygen

## Abstract

Reduction-oxidation (redox) reactions, in which electrons move from a donor to an acceptor, are the functional heart of photosynthesis. It is not surprising therefore that reactive oxygen species (ROS) are generated in abundance by photosynthesis, providing a plethora of redox signals as well as functioning as essential regulators of energy and metabolic fluxes. Chloroplasts are equipped with an elaborate and multifaceted protective network that allows photosynthesis to function with high productivity even in resource-limited natural environments. This includes numerous antioxidants with overlapping functions that provide enormous flexibility in redox control. ROS are an integral part of the repertoire of chloroplast signals that are transferred to the nucleus to convey essential information concerning redox pressure within the electron transport chain. Current evidence suggests that there is specificity in the gene-expression profiles triggered by the different ROS signals, so that singlet oxygen triggers programs related to over excitation of photosystem (PS) II while superoxide and hydrogen peroxide promote the expression of other suites of genes that may serve to alleviate electron pressure on the reducing side of PSI. Not all chloroplasts are equal in their signaling functions, with some sub-populations appearing to have better contacts/access to the nucleus than others to promote genetic and epigenetic responses. While the concept that light-induced increases in ROS result in damage to PSII and photoinhibition is embedded in the photosynthesis literature, there is little consensus concerning the extent to which such oxidative damage happens in nature. Slowly reversible decreases in photosynthetic capacity are not necessarily the result of light-induced damage to PSII reaction centers.

## Highlights

• Reactive oxygen species (ROS) are produced in abundance by photosynthesis.
• ROS and antioxidants function in redox signal transduction that is important in chloroplast to nucleus communication.
• Some chloroplasts have specialized signaling functions that regulate epigenetic as well as genetic programming.
• Photoinhibition and slowly reversible decreases in photosynthetic capacity are not necessarily the result of light-induced damage to PSII reaction centers.

## 1 Introduction

Electron flow is the basis of life on earth, whether it is driven by light in photosynthesis or through chemical redox couples in respiration. The incorporation of photosynthesis into eukaryotic cells during evolution not only provided enormous benefits in terms of energy metabolism, but also significantly extended the cellular capacity for reduction/oxidation (redox) regulation and signaling. The incorporation of the photosynthetic electron transport chain appears to have been accompanied by a large expansion of protein cysteine thiols used to sense and regulate cellular redox state (Woehle et al., 2017). The operation of photosynthesis also resulted in a large increase in the production of reactive oxygen species (ROS), such as superoxide, hydrogen peroxide and singlet oxygen. The explosion of oxidative signals arising from photosynthesis, together with the significant expansion of the redox-sensitive proteome, not only provided cells with mechanisms to monitor photosynthetic electron flow and thus prevent over-reduction or over-oxidation, but also produced redox regulatory networks that enable plants to sense and respond to fluctuating environmental conditions (Martin and Sies, 2017). While the concept that cells need to manage ROS production and removal to avoid excessive and irreversible oxidation is embed in the animal and plant literature, the consensus of opinion is now shifting towards a recognition of the positive roles of ROS as essential pro-life signals (Noctor and Foyer, 2016; Foyer et al., 2017). Within the complex redox landscape of photosynthetic cells, ROS production is essential for redox sensing, signaling and regulation. The best characterized signaling mechanisms involve redox changes in cysteines either on low-molecular-weight thiols, particularly glutathione, or on proteins thiol groups with oxidation states of the sulphur moieties ranging from thiols (–RSH) and disulfides (–RSSR–) to trisulfides and possibly even tetrasulfides (Noctor et al., 2011). In photosynthesis, this thiol-disulphide exchange system is used to co-ordinate the rates of NADP and ATP generation to the rates of their utilization in photosynthetic carbon assimilation through the mediation of thioredoxins (TRXs). This extensive network of interactive redox regulation occurs together with other post-translational modifications (PTM), particularly protein phosphorylation/dephosphorylation reactions that also serve to co-ordinate processes occurring in the thylakoids and stroma. Exposure to oxidative stress influences two other poorly characterized plant PTMs, protein succinylation and acetylation (Zhou et al., 2017). This analysis of H_2_O_2_-triggered interactions between the rice leaf lysine succinylome and acetylome revealed specific effects on photosynthetic proteins, but the functional significance of these PTMs remains to be established (Zhou et al., 2017). Moreover, the chloroplast thylakoid lumen also contains redo-regulated proteins. Within the lumen, proteins such as the immunophilin, FKBP13, polyphenol oxidase and violaxanthin de-epoxidase, are regulated by oxidative activation facilitated by the oxygen produced by PSII (Buchanan & Luan, 2005).

Light-driven redox changes in the photosynthetic electron transport system drive energy conversion that is harnessed in the forms of reducing power (reduced ferredoxin and NADPH) and ATP. The rate of carbon assimilation is matched to prevailing electron fluxes through the reduction of TRXs. Reduced TRXs then reduce disulphide bridges on target proteins within the Calvin cycle (Buchanan and Balmer, 2005; Nikkanen and Rintamäki, 2014). Chloroplasts house a large number of different TRX forms (Meyer et al., 2012). These TRXs can be reduced by two pathways that show some overlap with regard to their target proteins (Nikkanen and Rintamäki, 2014; Nikkanen et al., 2017). The ferredoxin-dependent TRX system is driven by reduced ferredoxin produced by PSI. Reduced TRX is generated through the mediation of ferredoxin-thioredoxin reductases. The pathway is responsible for the light-induced activation of enzymes of photosynthetic carbon assimilation (Michelet et al., 2013; Geigenberger and Fernie, 2014), including fructose-1,6-bisphosphatase (FBPase) and sedoheptulose-1,7-bisphosphatase (SBPase), phosphoribulokinase (PRK) and glyceraldehyde-3-phosphate dehydrogenase (GAPDH). In addition, this pathway regulates the thylakoid chloroplast ATP synthase (Hisabori et al., 2013), the malate-oxaloacetate shuttle (Scheibe and Dietz, 2012), the ADP-glucose pyrophosphorylase step of starch synthesis (Thormählen et al., 2013) and the Mg-protoporphyrin methyltransferase step in chlorophyll biosynthesis (Richter et al., 2013). In addition to the ferredoxin-dependent TRX system, there is an NADPH-dependent thioredoxin reductase (NTRC) pathway, which can also activate the carbon assimilation enzymes either directly or through interaction with the ferredoxin-TRX system. The operation of the NTRC pathway may be crucial in activating the photosynthetic enzymes under low light intensities (Nikkanen et al., 2017).

**Fig. 1:**
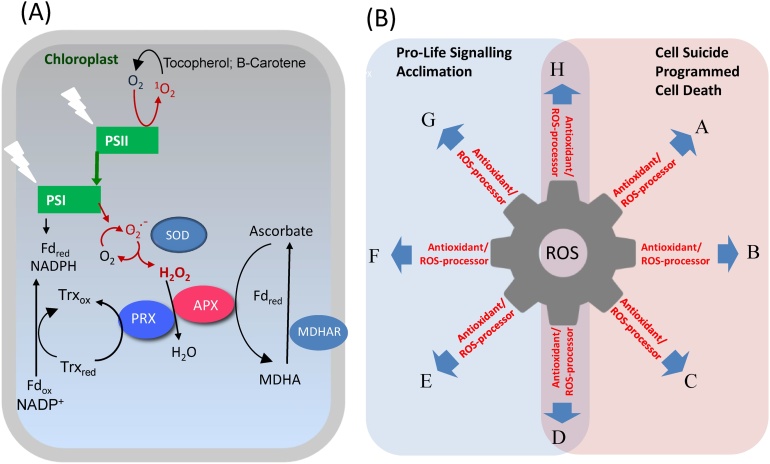
A. ROS formation and metabolism in chloroplasts. The chloroplast produces singlet oxygen at Photosystem (PS) II, whereas reduction of molecular oxygen to superoxide occurs predominantly at PSI. Hydrogen peroxide is produced from superoxide in a reaction catalyzed by superoxide dismutase (SOD). Hydrogen peroxide can be reduced to water by the action of ascorbate peroxidases (APX) or 2-Cys peroxiredoxins (PRX). Oxidation of ascorbate generates monodehydroascorbate (MDHA), which can be reduced back to ascorbate either by reduced ferredoxin or monodehydroascorbate (reductase (MDHAR). Make consistent between figure and legend] Oxidized PRX are reduced again by the action of thioredoxins (TRX). B. Current model for ROS-antioxidant interplay in cell signaling showing that antioxidants act as ROS-processing and –signaling mediators, allowing different options (for signal transduction. Letters indicate different possible pathways that are not mutually exclusive. The model indicates that loss of any one of these antioxidant components would drive processing and signaling through the other pathways..

The ferredoxin-dependent TRX and NTRC systems function together with 2-cysteine peroxiredoxins (2-Cys PRX) as a two-component hydrogen peroxide-detoxifying system under stress conditions (Perez-Ruiz et al., 2006; Bernal-Bayard et al., 2014). 2-Cys PRXs act together with the ascorbate-glutathione cycle to limit the accumulation of hydrogen peroxide produced by photosynthesis (Foyer and Shigeoka, 2011). It is also worthy of note that TRXz is required for the correct assembly of the plastid PEP polymerase and the developmental transition from nuclear-encoded RNA polymerase-driven transcription to PEP-dependent transcription of plastid genes, such as psbA that encodes the D1 protein of PSII (Dietz and Pfannschmidt, 2011). Moreover, TRXx, TRXy and CDSP32 are important in chloroplast responses to oxidative stress (Collin et al., 2003, Collin et al., 2004; Broin et al., 2002). ROS accumulation within chloroplasts is controlled by a complex antioxidant-scavenging system (Foyer and Shigeoka, 2011) that includes TRXs and 2-Cys PRXs as well as antioxidant enzymes, such as superoxide dismutase (SOD) and ascorbate peroxidase (APX), and low molecular weight antioxidants, such as ascorbate (Fig. 1A) that also play roles in transmitting oxidative signals as well as controlling ROS accumulation (Foyer et al., 2017; Noctor et al., 2017).

For many years, the dominant concept in redox signaling has been that cells balance ROS on one side and antioxidants on the other (Noctor et al., 2017). According to this concept, oxidative signaling shifts this balance such that ROS accumulate, either through an increase in their production or a decrease in antioxidant capacity. This concept suggests that, when ROS are low, cell signaling entrains acclimation and improved stress tolerance and that, when ROS are in excess of antioxidant capacity, the resulting enhanced oxidation causes damage and entrains programmed cell death. While this concept continues to be useful, recent work suggests that it needs to be updated, notably to take account of the complexity and specificity of plant antioxidative systems as well as their roles in signaling (Fig. 1B). It has, therefore, been suggested that “ROS processing systems” would be a more accurate term than “antioxidative systems” to describe cellular components that are most likely to interact with ROS and, in doing so, transmit oxidative signals (Noctor et al., 2017). The direction of signal flow will depend on factors such as the proximity of the ROS target, its relative susceptibility to oxidation and its abundance in relation to other targets. In this way, oxidative signals may have a high level of specificity in a ROS-determined wheel of cell fortune or fate. Within this context, redox signals arising from the chloroplasts will readily interact with components of the phytohormone signaling network to regulate plant growth and defense pathways in response to a changing environment (Bartoli et al., 2013).

## 2 ROS formation in chloroplasts

**Fig. 2:**
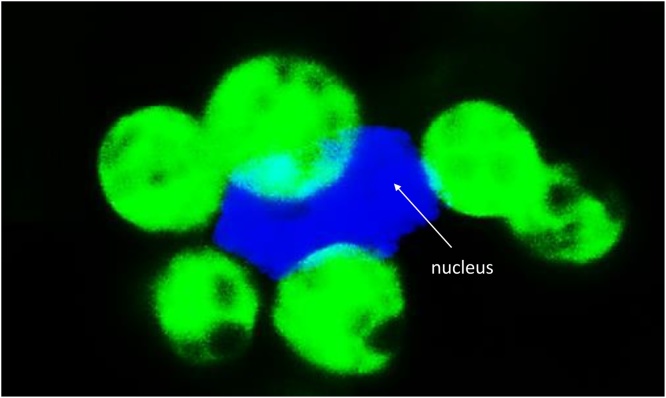
The close association of some chloroplasts with the nucleus (stained blue with DAPI) in *Arabidopsis thaliana* cotyledons, in which chloroplasts are labelled with green fluorescent protein. (For interpretation of the references to colour in this figure legend, the reader is referred to the web version of this article.)

**Fig. 3:**
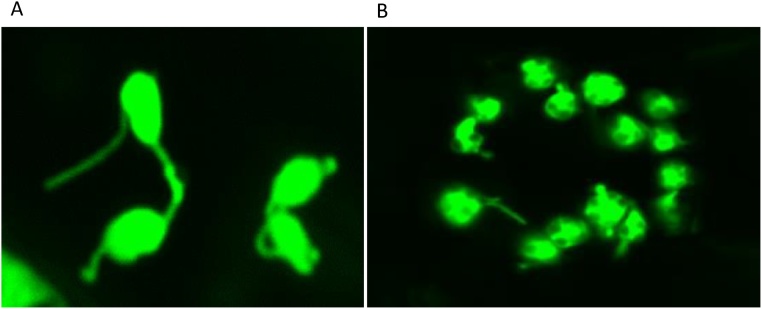
Chloroplast stromules in the mesophyll cells (A) and stomatal guard cells (B) of *Arabidopsis thaliana* cotyledons. Chloroplasts are labelled with green fluorescent protein. (For interpretation of the references to colour in this figure legend, the reader is referred to the web version of this article.)

Oxygen is an important electron acceptor in photosynthesis. The reduction of molecular oxygen by PSI in the Mehler reaction is a coupled process leading to the formation of ATP without NADPH. Oxygen reduction provides an alternative electron sink and generates superoxide anion radicals that are converted to H_2_O_2_ by the action of the thylakoid copper/zinc SODs. H_2_O_2_ can then be reduced to water by chloroplast APXs and the PRXs (Awad et al., 2015). In the overall process, known as the water-water cycle, two electrons are used to produce H_2_O_2_ and two are required to metabolize H_2_O_2_ to water, allowing the dissipation of excess excitation energy and electrons (Foyer et al., 1991; Osmond and Grace, 1995; Asada, 2000). While this process may only make a minor contribution to thylakoid acidification and the control of PSII activity because it is saturated at relatively low irradiances (Heber, 2002; Driever and Baker, 2011), it is undoubtedly an important source of oxidative signals that may be directly or indirectly be transferred to the nucleus (Fey et al., 2005; Noctor and Foyer, 2016; Exposito-Rodriguez et al., 2007). One possibility is that H_2_O_2_ generated by photosynthesis may be directly transferred to the nucleus from attached chloroplasts (Fig. 2; Exposito-Rodriguez et al., 2007). Thus, the Mehler reaction might itself function as a signaling mechanism, with H_2_O_2_ production acting as a measure of electron transport activity (Exposito-Rodriguez et al., 2007). Another mechanism of direct transfer of H_2_O_2_ to the nucleus is via stromules (Fig. 3; Caplan et al., 2015).

It is now well-established that ROS are key components of chloroplast‐nucleus retrograde signaling pathways, working together with a complex array of putative retrograde signaling molecules including metabolites and mobile transcription factors, calcium and links to pathways such as the unfolded protein response. Of these, the 3′-phosphoadenosine 5′-phosphate (PAP) phosphatase called SAL1 is perhaps the best characterized; SAL1 is thought to act as a sensor of oxidative stress in the chloroplasts because it undergoes a conformational change in response to redox changes. Chloroplast oxidation results in deactivation of SAL1 and accumulation of its substrate PAP, which acts as plastid signal instigating chloroplast-to-nucleus retrograde signaling pathways (Chan et al., 2016). It should be noted that redox changes in other cellular compartments may influence the susceptibility of photosynthesis to high-light-induced inhibition and also modulate chloroplast-to-nucleus signaling and the transcriptional responses of leaves to high light (Karpinska et al., 2017a, Karpinska et al., 2017b). Superoxide and H_2_O_2_ are relatively weak oxidants. Superoxide has received little attention with regard to chloroplast processes because it has no reactivity to most biological molecules and is rapidly converted to H_2_O_2_ by the action of thylakoid and stromal SODs. Superoxide interacts with nitric oxide (NO) and it is also reduced to H_2_O_2_ by ascorbic acid, which is abundant in chloroplasts. In the mitochondria, superoxide is able to inactivate the tricarboxylic acid cycle enzyme aconitase, which is a sensitive target in the matrix, but it has not been shown to cause inhibition of Calvin cycle enzymes that, in contrast, are very susceptible to oxidation and inactivation by H_2_O_2_. Hence, plastid H_2_O_2_ levels have to be very low and in the micromolar range. It is worthy of note that H_2_O_2_ and superoxide have divergent effects on cell survival and programmed cell suicide pathways in animals, where H_2_O_2_ promotes and superoxide inhibits cell death programs and apoptotic signaling (Pervaiz and Clement, 2007). It may be that superoxide also promotes cell survival in plants, particularly in light of observations of spatiotemporal patterns superoxide and H_2_O_2_ in root meristems (Foyer et al., 2018). The short lifetimes of superoxide and singlet oxygen make them unlikely candidates to diffuse over any distances within the cell. In contrast, H_2_O_2_ is more stable and is thus the most likely ROS form to act as a mobile signal (Foyer and Noctor, 2016).

Chloroplast ROS are essential signals underpinning acclimation responses through retrograde signaling to the nucleus, and they are also components of whole-plant systemic signaling pathways. The small plastids of the vascular parenchyma and also bundle-sheath plastids fulfil signaling functions that regulate mesophyll plastid development (Lundquist et al., 2014; Petrillo et al., 2014). Phytochrome B-mediated auxin pathways transmit signals perceived by apical leaves to increase the speed of induction of photosynthesis in distal leaves (Guo et al., 2016). This pathway, which transmits information concerning high light perceived at the apex, results in stimulation of H_2_O_2_ production in systemic leaves that are distant from the apical leaves [?] leading to increased cyclic electron flow around PSI during photosynthetic induction (Guo et al., 2016). Moreover, altering the ascorbate/DHA ratio of the apoplast/cell wall compartment by manipulating ascorbate oxidase activities was sufficient to alter the ability of photosynthesis to acclimate to high light (Karpinska et al., 2017a). Tobacco leaves with low ascorbate oxidase activities maintained higher photosynthesis rates under high light than wild type controls or plants with high ascorbate oxidase activities (Karpinska et al., 2017a). Taken together, such findings demonstrate that photosynthesis is not only responsive to ROS produced directly in the chloroplasts, but also to oxidants generated in other parts of the cell or via phytohormone-mediated pathways to allow acclimation to local and systemic cues (Bartoli et al., 2013).

In contrast to H_2_O_2_, singlet oxygen (^1^O_2_), is a powerful oxidant that reacts rapidly with macromolecules in the vicinity of where it is generated, resulting in oxidation that is often referred to as “damage” (Apel and Hirt, 2004; Watabe et al., 2007). Much of the oxidative inactivation, caused by over-excitation of the photosynthetic electron transport chain is caused by ^1^O_2_ (Telfer et al., 1999; Telfer, 2014; Krieger-Liszkay et al., 2008). The vulnerability of PSII to oxidative damage/inactivation means that each reaction center has to be rebuilt several times an hour, even under optimal irradiances. Oxidative modifications to the D1 and D2 proteins increase reaction center turnover (Malnoë et al., 2014). It is possible that the breakdown products of D1 protein might fulfil signaling roles, but this has not as yet been demonstrated in higher plants. The subunits of the oxygen-evolving complex are also prone to ROS- mediated damage/modification (Henmi et al., 2004). Any damaged/inactivated subunits are removed by luminal proteases that degrade damaged/inactivated components during the repair cycle, in which newly synthesized subunits are shuttled from the stroma lamellae, where they are inserted into the thylakoids, to the granal thylakoids. Singlet oxygen can also impair *de novo* synthesis of D1 by targeting the protein elongation factor G (CpEF-G) (Nishiyama et al., 2006). D1 is co-translationally inserted into PSII during repair via the cpSECY translocase (Nilsson and van Wijk, 2002). However, ^1^O_2_ would have to diffuse over a considerable distance if it were to target the elongation factors, and there are many other susceptible molecules in the thylakoid system, particularly poly-unsaturated fatty acids (PUFA) and other membrane lipids (Przybyla et al., 2008). These peroxidation products are generated in a singlet oxygen-dependent fashion in plants exposed to high light and other stresses (Triantaphylidès et al., 2008). Lipid peroxidation, accompanied by the generation of oxylipins, is often taken to be a signature of photo-oxidative damage (Girotti, 2001; Mueller et al., 2006; Przybyla et al., 2008). Moreover, excessive lipid peroxidation, leading to the apparent bursting of the chloroplast membranes, is often considered by the basis for the “accidental cell death” phenomena described in mutants such as *flu* and *ch1* (Laloi and Havaux, 2015). However, chloroplasts contain many ^1^O_2_ scavengers, including β-carotene ascorbate, tocopherol and glutathione that prevent such negative outcomes. Moreover, photo-protective mechanisms are deployed in order to protect the photosynthetic antenna complexes from over-excitation, when the absorption of light intensity exceeds the capacity of photochemistry to use the absorbed energy.

Superoxide and hydrogen peroxide are largely generated at PSI on the stromal side of non-appressed thylakoid membranes. Singlet oxygen is generated by PSII in the grana core and also by the PSII repair process occurring on non-appressed margins of the grana (Wang et al., 2016). Accumulating evidence suggests that hydrogen peroxide and singlet oxygen function as separate redox signals that are transduced by different pathways to trigger the expression of specific suites of nuclear genes. Two distinct ^1^O_2_ −triggered chloroplast-to-nucleus signaling pathways have been described to date: firstly, ^1^O_2_ may modify gene expression through interaction with β-carotene and the generation of carotenoid breakdown products (β-CC), some of which are volatile (Ramel et al., 2012); secondly, the nuclear-encoded chloroplast protein EXECUTER (EX)1 plays an essential role together with EX2 in the transmission of ^1^O_2_ signals (Apel and Hirt, 2004; Lee et al., 2007, op den Camp et al., 2003; Wagner et al., 2004, Wang et al., 2016). The β-CC-induced and EX1-dependent pathways have only a small number of common ^1^O_2_ −responsive genes, suggesting that the two pathways are separate (Dogra et al., 2017). Moreover, β-CC-mediated signaling is independent of EX1/EX2 (Ramel et al., 2013; Shumbe et al., 2016). The EX1 and EX2 proteins are required for the local and systemic signaling pathways leading to gene expression changes that underpin acclimation to stress caused by high light (Carmody et al., 2016). The EX1 proteins are mostly localized in the non-appressed margins of the grana, suggesting that EX1 transmits signals from ^1^O_2_ produced in the grana margins (Wang et al., 2016), where the repair of damaged/inactivated PSII reaction centers (RCII) takes place through disassembly and degradation of damaged/inactivated proteins by the membrane-bound FtsH protease. EX1 is associated with RCII proteins, including D1 and D2, and FtsH2 proteases, as well as with protein-elongation factors and chlorophyll biosynthetic enzymes (Wang et al., 2016). In contrast, β-carotene is likely to be more important in quenching ^1^O_2_ produced in the grana core (Ramel et al., 2012, Ramel et al., 2013).

Perception of ^1^O_2_ signals induces cell suicide programs in seedlings and leads to growth inhibition in mature plants (Wagner et al., 2004). Phytohormones such as salicylic acid and jasmonate act as important promoters or inhibitors of cell death in the *fluorescent* (*flu*) and *chlorina 1* (*ch1*) mutants, acting as key players in the decision between acclimation and cell death as they do in H_2_O_2_–mediated induction of programmed cell death observed in *catalase* (*cat)2* mutants (Mhamdi et al., 2010, Mhamdi et al., 2012). The FLU protein acts as a negative feedback regulator in the Mg-branch of the tetrapyrrole biosynthetic pathway in the dark. The *flu* mutants accumulate the chloroplast precursor protochlorophyllide (Pchlide) in the dark because conversion of Pchlide to Chlorophyllide is light-dependent. Pchlide molecules are potent photosensitizers that generate ^1^O_2_ in abundance upon illumination (Meskauskiene et al., 2001).

## 3 Photodamage and photo-protection

Photosynthesis converts light energy into chemical energy through an interacting series of biophysical and biochemical reactions that provide the essential driving force for reductive metabolism in plants. The dynamic regulation of the photosynthetic machinery allows continuous adjustments to changes in the availability of light and carbon dioxide. Plants cannot easily turn off photosystem chemistry and this flexibility is thus crucial, not least because PSII is extremely sensitive to light-induced oxidative inactivation (Aro et al., 1993; Yokthongwattana and Melis, 2006). PSI is also susceptible to light-induced inhibition, particularly at low temperatures or under high light, and this has consequences for photosynthetic carbon fixation and cell signaling (Gollan et al., 2017). Photosynthetic regulation is designed to protect the system, while maintaining the balance between energy-producing and energy-consuming processes. Optimizing the efficiency of photosynthesis is a priority when light is limiting, allowing effective use of solar power and ensuring that metabolism is not limited by energy supply. Since light is a potentially dangerous substrate, light use must be carefully managed.

Electron transport and formation of ATP and NADPH proceed at much slower rates than light harvesting and energy transfer to RCII by antennae (Ruban et al., 2012). As an immediate consequence, the amount of energy absorbed recurrently overcomes the metabolic energy demands. This leads to accumulation of excess energy in the thylakoid membrane that can potentially be harmful to PSII, leading to the permanent closure of the PSII reaction centers and photoinhibition, which can be defined as a sustained depression of Fv/Fm ratio (Aro et al., 1993). The probability of light-induced inactivation of RCII increases when Q_A_ is reduced, i.e., when electron transport away from PSII is limited. However, the PSII reaction centers are protected by mechanisms that diminish the rate of excitation of PSII by harmlessly dissipating excess energy in the PSII antenna complexes as heat. This protection is quantified by processes that can be assessed from non-photochemical quenching of chlorophyll fluorescence (NPQ). Several processes that contribute to NPQ can be distinguished through their recovery kinetics. These are the state transition (qT), photoinhibition (qI), heat dissipation (qE) and zeaxanthin-dependent (qZ) quenching (Baker, 2008; Jahns and Holzwarth, 2012; Ruban et al., 2012). Although these NPQ components are kinetically distinct, they have overlapping quenching mechanisms and functions (Logan et al., 2014; Demmig-Adams et al., 2014). The pH-dependent NPQ component that is typically rapidly reversible in the dark can be maintained in darkness under conditions that maintain trans-thylakoid pH in darkness. Similarly, the zeaxanthin-correlated NPQ component can be reversed in minutes in some circumstances but can be maintained for days or weeks in darkness under photoinhibitory conditions. The qT component is associated with redistributing energy absorption between the two photosystems, which is achieved by the partial detachment and migration of LHCII between PSII and PSI (Bellafiore et al., 2005). The qI component is a slowly reversible process and the consequence of photoinhibitory events reflecting inactivation of the PS II reaction centres, where closed PS II reaction centres protect the open PS II reaction centres (Matsubara and Chow 2004). The major and fastest component of NPQ, qE, is believed to act in RCII photoprotection, and is related to the dissipation of excess energy as heat (Baker, 2008; Ruban et al., 2012). More recently described and with a formation/relaxation lifetime in the order of 10–15 min, qZ is believed to be correlated with the synthesis and epoxidation of zeaxanthin, and is also related to photoprotection (Nilkens et al., 2010; Jahns and Holzwarth, 2012). The site of qE formation is believed to be the light-harvesting antenna of PSII, triggered by ΔpH and enhanced by violaxanthin de-epoxidation to zeaxanthin (Horton and Ruban, 1992). The protein PsbS was discovered to play a crucial role in sensing lumen acidification and transducing the signal to the antenna. Although NPQ is the most commonly encountered form of PSII regulation, other mechanisms may also be important (Demmig-Adams and Adams, 2006). However, such assessments are complicated because multiple pathways contribute to photoprotection and these operate over different timescales. For example, the pH-dependent NPQ component that is typically rapidly reversible in the dark can be maintained in darkness if a trans-thylakoid pH persists (Gilmore and Björkman, 1995). Similarly, the zeaxanthin-correlated NPQ component can be reversed in minutes in some circumstances and can be maintained for days or weeks in darkness under photoinhibitory conditions (Demmig-Adams et al., 2012). Thus, it is not trivial to measure or quantify the different NPQ components accurately and this has led to a lack of consensus concerning the molecular mechanisms involved (Aro et al., 1993; Jahns and Holzwarth, 2012; Ruban et al., 2012; Tyystjärvi, 2013). A new method of NPQ measurement was proposed by Ruban and Murchie (2012) that involves determination of fluorescence responses to a gradually increasing actinic light routine. The yield of chlorophyll fluorescence can then be tracked to provide a measure of the onset of photo-inactivation (Ruban and Murchie, 2012). While sustained NPQ formation and zeaxanthin retention have been typically associated with the degradation of D1 degradation and other PSII proteins in overwintering conifers and other evergreens (Demmig-Adams et al., 2014), photoinhibitory damage may not be a common phenomenon in nature (Foyer et al., 2017). Within this context, photoinhibition may be consequence, rather than a cause, of limited productivity (Adams et al., 2013). Stress-induced sustained decreases in Fv/Fm ratios are more likely to be related to PSII downregulation via sustained components of protective NPQ and hence should not be used as a measure of damage resulting from photoinhibition (Foyer et al., 2017). Moreover, recent evidence shows that leaves adjust photosynthetic efficiency in response to changing irradiance relatively slowly. For example, wheat leaves were shown to take 15 min to regain maximum photosynthetic efficiency following transfer from shade to sun conditions, the major limitation being the activation of ribulose-1, 5-bisphosphate carboxylase/oxygenase (Taylor and Long, 2017). Moreover, decreasing the time required for NPQ relaxation was found to increase the efficiency of CO_2_ assimilation in tobacco leaves such that productivity was increased by up to 20% (Kromdijk et al., 2016). The observed enhancement of biomass production may not only be due to increased energy availability as a result of an improved efficiency of photon capture, but also to an increase in the production of ROS signals that stimulate cell expansion and growth (Demmig-Adams et al., 2018). An association between increased growth, decreased thermal dissipation and enhanced ROS production is consistent with findings of Esteban et al. (2009).

## 4 Chloroplast antioxidants

Chloroplasts are armed with an elaborate arsenal of antioxidants that often have overlapping or interacting functions. The function of chloroplast antioxidants is not to totally eliminate superoxide, H_2_O_2_ and ^1^O_2_, but rather to achieve an appropriate balance between production and removal that is compatible with the operation of photosynthesis and yet allows effective transmission of these redox signals to the nucleus. Carotenoids such as lutein and zeaxanthin are effective thylakoid antioxidants located in close proximity to chlorophylls in the light-harvesting complexes. These pigments are able to quench the triplet state of chlorophyll (3Chl*) and ^1^O_2_, resulting in a higher-energy triplet state (Car*), which can decay to the ground state via different reaction mechanisms such as intersystem crossing, triplet-triplet annihilation and ground state quenching (Burke et al., 2000). The PSII reaction center contains two β-carotene molecules (Ferreira et al., 2004), which are positioned too far from the reaction centre special pair, P680* to allow direct quenching. However, when ^1^O_2_ is generated by P680*, the distance to the β-carotene is close enough to allow oxidative modification of β-carotene and signaling (Ramel et al., 2012). Tocopherols are also important membrane ^1^O_2_ quenchers, which function as general protectors of thylakoid membranes against lipid peroxidation (Piller et al., 2014). These membrane-bound antioxidants are oxidized either in a one-electron-transfer reaction to a tocopheryl-radical or are oxidized ^1^O_2_ to a hydroperoxide. Both reactions can be reversed by ascorbate, which can reduce both the radical and hydroperoxide (Krieger-Liszkay et al., 2008). The α − tocopherols in the thylakoid membranes are also able to terminate chain reactions of PUFA free radicals generated by lipid oxidation. Lipid alkoxyl and peroxyl radicals are reduced to alcohols or hydroperoxides, and chromanoxyl radicals, which are all less efficient in propagation of lipid peroxidation. The chromanoxyl radical may be reduced by ascorbate, thereby regenerating α − tocopherol molecule. While Arabidopsis mutants lacking tocopherol cyclase (*vte1***)** showed little increase in stress sensitivity compared to the wild type, crossing *vte1* with the *npq1* mutant that is deficient in violaxanthin deepoxidase, the enzyme responsible for the conversion of violaxanthin to zeaxanthin in the thylakoid membrane, resulted in oxidation of lipids and pigments, as well as photoinhibition (Havaux et al., 2005). In addition to its functions in NPQ, zeaxanthin can function directly as an antioxidant.

Chloroplasts contain a number of pathways that limit H_2_O_2_ accumulation_,_ including the ascorbate-glutathione cycle (Foyer and Shigeoka, 2009). In its basic form, this pathway consists of a network of reactions involving APX and enzymes that thereafter serve to regenerate ascorbate. APX produces monodehydroascorbate radicals (MDHA), which can then be reduced back to ascorbate by ferredoxin or by NAD(P)H in a reaction catalysed by MDHA reductase (MDHAR; Fig. 1A). MDHA that escapes these reactions can spontaneously disproportionate to ascorbate and dehydroascorbate (DHA). DHA must then be recycled to ascorbate in order to avoid depletion of ascorbate. In chloroplasts, reduced glutathione (GSH) can reduce DHA non-enzymatically or via the enzyme DHA reductase (DHAR), in a reaction in which GSH is oxidized to glutathione disulfide (GSSG). However, an analysis of Arabidopsis *dhar* mutants, including DHAR3 that has a chloroplastic location, showed that DHAR activity can be decreased to negligible levels without marked effects on the leaf ascorbate pool (Rahantaniaina et al., 2017). Intriguingly, the *dhar1 dhar2* double mutants showed decreased GSH oxidation in a *cat2* background with inhibition of *cat2*-triggered induction of the salicylic acid and cell death pathways. However, these effects were reversed in the *cat2 dhar1 dhar2 dhar3* mutant, suggesting the presence of complex redox signaling pathways that remain to be elucidated (Rahantaniaina et al., 2017). Recycling of GSSG to GSH is catalysed by glutathione reductase (GR) using NADPH produced by the thylakoid electron transport chain.

Analysis of Arabidopsis mutants lacking both stromal (sAPX) and thylakoid (tAPX) forms of APX, however, revealed that the chloroplasts do not depended on the ascorbate-glutathione cycle alone to remove H_2_O_2_ (Giacomelli et al., 2007; Kangasjärvi et al., 2008; Maruta et al., 2010). In contrast, mutants lacking the thylakoid 2-Cys PRXs (*2cpa 2cpb*) had impaired photosynthetic efficiency and showed signs of oxidation suggesting that the chloroplast 2-Cys PRXs are essential and perhaps more important than tAPX in preventing H_2_O_2_ accumulation. However, 2-Cys PRXs are also involved in the reduction of reactive lipid peroxides, thus providing additional protection to the chloroplasts. Interestingly, while the 2cpa 2cpb tapx triple mutants showed oxidative stress markers including expression of H_2_O_2_-responsive marker genes, leaf H_2_O_2_ levels were similar to those measured in the 2cpa 2cpb (Awad et al., 2015). These findings suggest that APXs work together with the 2-Cys PRXs and perhaps other systems to remove H_2_O_2_ produced by the photosynthetic electron transport chain and keep stromal H_2_O_2_ levels low (Awad et al., 2015).

While the mechanisms by which ascorbate is transported into chloroplasts remain to be fully characterized, a member of the phosphate transporter 4 family (AtPHT4;4) was shown to transport ascorbate across the envelope membrane (Miyaji et al., 2015) and at least one member of the nucleobase ascorbate transporter (NAT) family may be localized on the thylakoid membrane. Knockout mutants lacking AtPHT4;4 were compromised in thermal energy dissipation (Miyaji et al., 2015). In the thylakoid membrane, ascorbate can act as an alternative electron donor to PSII, and it is also required for the conversion of violaxanthin to zeaxanthin in the xanthophyll cycle (Müller-Moulé et al., 2003; Jahns et al., 2009). Violaxanthin deepoxidase (VDE) is a luminal enzyme that is active only in its completely oxidized form that has six disulfide bonds. It is, therefore, possible that this enzyme is regulated by dithiol/disulfide exchange reactions (Simionato et al., 2015). The VDE reaction has a low affinity for ascorbate. Moreover, the affinity of the latter enzyme for ascorbate is strongly pH-dependent (Bratt et al., 1995; Jahns et al., 2009). Since chloroplasts contain about 10 mM ascorbate (Zechmann et al., 2011) and VDE requires very high levels of ascorbate for saturation e.g. 100 mM at pH 6.0 (Bratt et al., 1995; Jahns et al., 2009), it is highly unlikely that these levels are achieved within the thylakoid lumen. The granal thylakoid lumen significantly expands in the light, allowing better movement of plastocyanin and alleviating restrictions imposed on protein diffusion and the transport of electrons both to and from the cytochrome b6f complex in this compartment in the dark (Kirchhoffa et al., 2011). Nevertheless, measured levels of ascorbate within the thylakoid lumen are much lower than those measured in the stroma (Zechmann et al., 2011; Heyneke et al., 2013). Hence, VDE activity must always be limited by ascorbate availability in the wild type even under optimal conditions. The Arabidopsis *vtc2-1* mutants, which have low ascorbate, showed higher levels of photoinhibition and photooxidation than the wild type under high light (Müller-Moulé et al., 2003, Müller-Moulé et al., 2014). However, other antioxidants such as glutathione are increased to compensate for low ascorbate and maintain antioxidant capacity (Pastori et al., 2003; Pavet et al., 2005).

Adaptation to biotic and abiotic threats in the environment is thought to have a long-lasting impact on plant processes that is passed to successive generations through persistent epigenetic mechanisms that provide a pre-emptive advantage. While a growing body of evidence suggests that changes in the reduction/oxidation (redox) status of stress signaling molecules and the level of DNA methylation play a major role in the transgenerational embedding of stress tolerance, little information is available concerning the specific pathways and mechanisms involved. The plant’s environmental sense and response mechanism contains a proteome distinct but overlapping with that of the photosynthetic chloroplast, with several stress proteins, including MSH1, appearing uniquely localized to the sensory plastid.

## 5 Chloroplast pathways contribute to epigenetic stress memory in plants

Chloroplasts are important sensors of environmental change, producing redox and other signals that can be transferred directly, or indirectly, to the nucleus to influence gene expression leading to acclimation to prevailing environmental conditions. Redox signals arising in chloroplasts also participate in epigenetic controls (Dietzel et al., 2015). Recent evidence suggests the presence of specific sub-sets of chloroplasts that specialize in sensory/signaling functions and participate in epigenetic reprogramming (Virdi et al., 2016). This sensory role is highlighted by analysis of the functions of MUTS HOMOLOG1 (MSH)1, a nucleoid protein that binds DNA and localizes to plastid and mitochondrial nucleoids (Xu et al., 2012; Virdi et al., 2016). The MSH1 protein, which is unique to plants, is targeted to both the mitochondria and chloroplasts and fulfils important functions in the induction of epigenetic stress memories in plants. MSH1-associated genetic reprogramming is a chloroplast-driven process accompanied by mitochondrial genomic and epigenetic responses that are intimately associated with organellar redox changes and stress responses (Virdi et al., 2016). Consequently, MSH1-containing organelles are considered to function as sensory plastids (Virdi et al., 2016).

Adaptation to the environment has both short term and long-lasting impacts on plant processes. In some cases, such stress memories are passed to successive generations through persistent epigenetic mechanisms that provide a pre-emptive advantage in a changing climate. Crucially, cellular redox status is a regulator of the level of DNA methylation, which plays a major role in the transgenerational embedding of stress tolerance memories in animals and plants. Reprogramming of the methylome i.e. the set of nucleic acid methylation modifications in the genome, predisposes the next generation for enhanced stress responses. The redox state of the sensory chloroplast population may also play a pivotal role in the regulation of such epigenetic controls in plants. The specialized sensory chloroplasts that house the *MSH1* pathway have been found to be localized in the epidermal and vascular parenchyma, as well as in reproductive cells. The sensory chloroplasts, which are about 30% of the size of photosynthetic chloroplasts and have decreased granal stacking, appear to be otherwise very similar to the more general chloroplast population, whose main function is photosynthesis, but they contain additional stress proteins, including MSH1, which associates with the thylakoid membrane and plastoglobuli (Virdi et al., 2016). Silencing *MSH1* produces a strong plant-stress response with differential expression of genes associated with abiotic and biotic stress pathways, together with genome-wide methylome re-patterning (Virdi et al., 2015). Subsequent segregation of the MSH1-RNAi transgene produced a trans-generational stress memory in 10-25% of progeny, with reduced growth, pale leaves, delayed maturity transition, delayed flowering, enhanced stress tolerance and altered circadian rhythms (Virdi et al., 2015). The *msh1* memory is stable and heritable indefinitely.

The sensory function of *MSH1* is clearly associated with the redox functions of the chloroplasts. It is possible that the MSH1 protein is able to move from the chloroplast to the nucleus, as has been suggested for another plastid single-stranded DNA-binding protein, WHIRLY1 (Grabowski et al., 2008). The partitioning of WHIRLY1 between chloroplasts and nuclei was shown to depend on phosphorylation by Calcineurin BLike‐Interacting Protein Kinase14 (CIPK14), providing a potential link between protein phosphorylation, Ca^2+^ signalling and WHIRLY1 functions (Ren et al., 2017). It is likely that these and other important multi-targeted and multifunctional organellar nucleoid proteins function in plastid-to-nucleus communication during developmental and stress responses, and perhaps passing directly from the chloroplasts to the nuclei (Caplan et al., 2015).

## 6 Conclusions and perspectives

The driving force for photosynthesis, sunlight, is an almost limitless supply. Hence, energy conservation is not a key issue for photosynthetic regulation. A sophisticated network of mechanisms that dissipate energy are essential to the operation of light harvesting linked to electron transport in photosynthesis, allowing effective protection against excess irradiance. The flexibility of photosynthesis has become optimized during evolution allowing photosynthesis to operate over a wide range of irradiances and environmental variables. The photosynthetic processes have evolved to be highly plastic and flexible, allowing other processes such as ROS production to occur at the expense of efficiency. Multiple layers of overlapping protective systems serve to prevent damage to the photosynthetic machinery, via combined action of preemptive energy dissipation and ROS removal through antioxidants. Give the requirement to perceive fluctuations in factors that influence the effective operation of photosynthesis in order to regulate these protective mechanisms, it is not surprising that the chloroplast became a major sensor of environmental and metabolic change with the ability to transmit retrograde signals to the nucleus in order to make appropriate adjustments in gene expression. Direct regulation of plastome gene expression is achieved via activation of protein kinases through the reduction state of photosynthetic electron transport chain components, notably plastoquinone and the cytochrome b_6_f complex. This regulation extends as far as regulation of nuclear alternative splicing to adjust plant responses to varying light conditions (Petrillo et al., 2014). This involves regulation of protein kinases located in the thylakoid membrane and in the stroma that, together with thioredoxin z, control the function of the plastid polymerase complex (Allen, 2015). While it is less clear how the redox state of the photosynthetic electron transport chain influences the expression of nuclear genes, ROS-related signals are established components of chloroplast-to-nuclear signaling. The importance of such communication pathways may have led to further specialization of certain sub-sets of chloroplasts to fulfil a crucial role in the epigenetic preservation of stress memories.

Despite compelling evidence that ROS are pro-life signals with multi-faceted roles in plant growth and development, the concept that oxidative damage is a major cause of light-induced loss of cellular functions remains prominent in the photosynthesis literature. This may be in part due to the heavy reliance on measured decreases in Fv/Fm ratios as a measure of photoinhibition, without consideration that such changes may be related to sustained PSII downregulation via protective systems. Hence, caution should be exercised in the use of Fv/Fm ratios to measure of photo-damage (Foyer et al., 2017).

Chloroplasts are equipped with an abundance of antioxidants that clearly have overlapping functions as ROS targets. Some of these antioxidants, such as glutathione and PRX, have well characterized roles in signal transduction. While others, such as ascorbate, may simply serve to attenuate the ROS signal, the complexity of the chloroplast oxidant and antioxidant system affords an extensive repertoire of discrete and probably specific redox signals that provides enormous flexibility in the control of gene expression. This communication with the nucleus not only conveys essential information on redox pressure within the electron transport chain to triggers appropriate short-term genetic responses, but also allows strategic provision of future defenses through epigenetic modifications.
